# Increased red blood cell distribution width might predict left ventricular hypertrophy in patients with atrial fibrillation

**DOI:** 10.1097/MD.0000000000022119

**Published:** 2020-09-11

**Authors:** Hui-Ming Yao, Xiao-Lei Wang, Xiong Peng, Shu-Yun Chen, Xuan Wan, Wei Zuo, Xin Gan

**Affiliations:** a Department of Respiratory and Critical Care Medicine, The First Affiliated Hospital of Nanchang University; b Second Department of Cardiovascular Medicine, Jiangxi Provincial People's Hospital Affiliated to Nanchang University, Nanchang, Jiangxi, China.

**Keywords:** atrial fibrillation, left ventricular hypertrophy, receiver operating characteristic, red blood cell distribution width, risk factors

## Abstract

The presence of left ventricular hypertrophy has been confirmed to be an independent risk factor for stroke and death in patients with atrial fibrillation. This retrospective study aimed to evaluate the potential risk factors for left ventricular hypertrophy in patients with atrial fibrillation.

A series of consecutive patients diagnosed with atrial fibrillation between June 2018 and December 2019 were included. The patients’ clinical data were analyzed. The cut-off values, sensitivity and specificity of the independent risk factors were calculated using a receiver operating characteristic curve.

Among 87 patients with atrial fibrillation, 39 patients with left ventricular hypertrophy and 48 patients without left ventricular hypertrophy were included. Multivariate logistic regression analysis showed that red blood cell distribution width (odds ratio [OR] 4.89, 95% confidence interval [CI]: 1.69–14.13, *P* < .05) was an independent risk factor, while the concentration of low-density lipoprotein (OR 0.37, 95% CI: 0.17–0.83, *P* < .05) and left ventricular ejection fraction (OR 0.88, 95% CI: 0.82–0.95, *P* < .05) were inversely associated with left ventricular hypertrophy in atrial fibrillation patients. The receiver operating characteristic curve demonstrated that the area under the curve was 0.80 (95% CI: 0.71–0.90, *P* < .05) with a cut-off value of 13.05, and the red blood cell distribution width predicted left ventricular hypertrophy status among atrial fibrillation patients with a sensitivity of 72.1% and a specificity of 76.9%.

Red blood cell distribution width was associated with left ventricular hypertrophy in patients with atrial fibrillation.

## 1 Introduction

Left ventricular hypertrophy (LVH) is a common type of target organ damage induced by cardiovascular disease, and the presence of LVH has been confirmed to be an independent risk factor for stroke and death in patients with atrial fibrillation (AF).^[1,2]^ Patients with AF lose atrial pump function, resulting in reduced cardiac output and hemodynamic deterioration. The comorbidity of LVH further exacerbates cardiac malfunction. Notwithstanding different threshold criteria and definitions, evidence suggests that 29.1% of AF patients have the complication of LVH.^[3]^ Identifying specific cardiac biomarkers for LVH in patients with AF has great potential for improving risk stratification and preventing adverse cardiovascular events.

As an index of the quantitative measure of the variability in the size of blood cells, red blood cell distribution width (RDW) is used in a heterogeneous cell population and is also a novel biomarker of morbidity and mortality associated with cardiovascular events.^[4,5]^ Recent studies indicate that increased RDW is associated with a higher possibility of long-term adverse events of cardiovascular diseases.^[6]^ Increased RDW level appear to be an independent predictor of left ventricular hypertrophy in patients with untreated essential hypertension.^[7]^ Inflammatory status is significantly related to ineffective red blood cells, which is partly reflected by an increase in RDW.^[8,9]^ Other mechanisms, including endothelial damage and oxidative stress, have been posited,^[10,11]^ but the exact pathophysiological underpinnings of the aforementioned associations remain controversial. Considering the above situation, we sought to evaluate whether RDW could be a potential predictor of LVH in patients with AF. However, to the best of our knowledge, the relationship between RDW and LVH in patients with AF has not been investigated previously. Therefore, the goal of the present study was to investigate the relationship between LVH and RDW levels in these patients.

## 2 Materials and methods

### 2.1 Patients

This was a retrospective study with no involvement of clinical or animal research and would not have any impact on the patient's prognosis. The requirement for ethical approval was waived according to the statements regarding ethical permission of the Ethical Committee of the First Affiliated Hospital of Nanchang University. A retrospective study was conducted, and a series of consecutive patients diagnosed with AF between June 2018 and December 2019 were included in this study. Patients were recruited if they were aged ≥18 years and had an AF diagnosis based on 12-lead electrocardiography or 24-hours Holter monitoring in the preceding 12 months. The exclusion criteria were as follows: patients with secondary AF; patients treated with catheter ablation and antiarrhythmic drugs for AF; patients with a history of hypertension over 10 years; patients with congenital heart disease such as congenital heart disease, valvular heart disease, cardiomyopathy, known coronary artery or cerebrovascular disease; disabling diseases; and inability to cooperate.

### 2.2 Data collection

All patients’ clinical data were recorded, and physical examinations and laboratory examinations, including echocardiographic measurements, were performed. Measurements of mean systolic blood pressure (SBP), mean diastolic blood pressure (DBP), high-density lipoprotein (HDL), low-density lipoprotein (LDL), total cholesterol, triglycerides, blood glucose, serum creatinine, uric acid, RDW, hemoglobin, interventricular septum thickness (IVST), left ventricular end-diastolic diameter (LVEDD), left ventricular posterior wall end-diastolic thickness (LVPWT), left ventricular end-diastolic left atrial diameter (LAD), and left ventricular ejection fraction (LVEF) were used in this analysis.

Echocardiogram recordings were performed by diagnostic medical sonographers in our hospital who used a parasternal window to record ≥10 consecutive beats of M-mode and 2-dimensional recordings of wall thicknesses and left ventricular internal diameter just below the mitral leaflet tips in short- and long-axis views. IVST, LVPWT, and LVEDD were used to calculate left ventricular mass (LVM) by a validated formula^[12]^: LVM = 1.04 × 0.8 × ([IVST+LVPWT+LVEDD]^3^–LVEDD^3^) + 0.6. Left ventricular mass index (LVMI) = LVM/BSA (body surface area, BSA). LVH was defined as follows: LVMI ≥110 g/m^2^ in women and ≥125 g/m^2^ in men.^[7]^

### 2.3 Statistical analysis

All analyses were performed using IBM SPSS Version 22 (SPSS Inc., Chicago, IL). Differences between qualitative variables are expressed as numbers and percentages and were tested by the chi-squared test or Fisher exact test. Continuous variables are reported as the means ± standard deviations and were tested with independent samples *t* tests or univariate analysis. Pearson correlation coefficients were calculated if appropriate. Independent risk factors related to LVH were calculated using a multivariate logistic regression analysis model. A receiver operating characteristic (ROC) curve was used to explore the predictive value of risk factors in patients with LVH. Statistical significance was indicated by a *P* value <.05.

## 3 Results

### 3.1 Clinical characteristics of study participants with and without LVH

The clinical characteristics of the study participants according to LVH status are shown in Table 1. Thirty nine patients with LVH and 48 patients without LVH were included in this study. Among the 87 patients with AF, 46 were men. In the comparisons of clinical characteristics, the mean SBP and LDL differed between patients with and without LVH (mean SBP: 133.41 ± 19.56 mmHg vs 121.3 ± 15.56 mmHg; 2.47 ± 0.83 mmol/dL vs 2.88 ± 0.72 mmol/dL, both *P* < .05). The patients with LVH had significantly higher RDW (13.45 ± 0.66 vs 12.80 ± 0.60, *P* < .05) and IVST levels (1.00 ± 0.09 cm vs 0.94 ± 0.11 cm, *P* < .05) than the patients in the non-LVH group. LVMI was significantly higher in the LVH group than in the non-LVH group (135.92 ± 11.71 g/m^2^ vs 95.53 ± 16.36 g/m^2^, *P* < .05). Moreover, the results showed that patients in the LVH group were characterized by a smaller LVPWT and LVEF than the non-LVH group (all *P* values < .05). Regarding sex, subtype of AF, mean DBP, HDL, total cholesterol, triglycerides, blood glucose, serum creatinine, uric acid, hemoglobin, LVEDD, and LAD, there were no statistically significant differences between the 2 groups (all *P* values >.05).

**Table 1:**
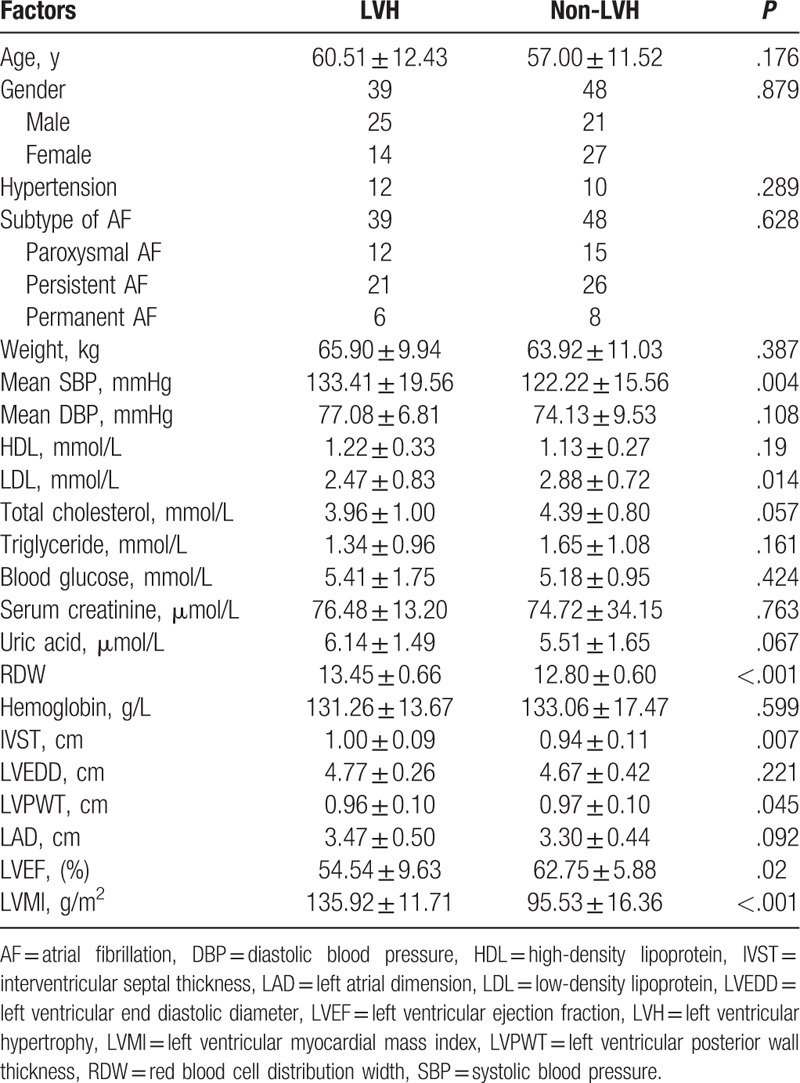
Clinical characteristics of participants with and without LVH.

### 3.2 Pearson correlation tests between RDW and other potential risk factors

**Figure 1:**
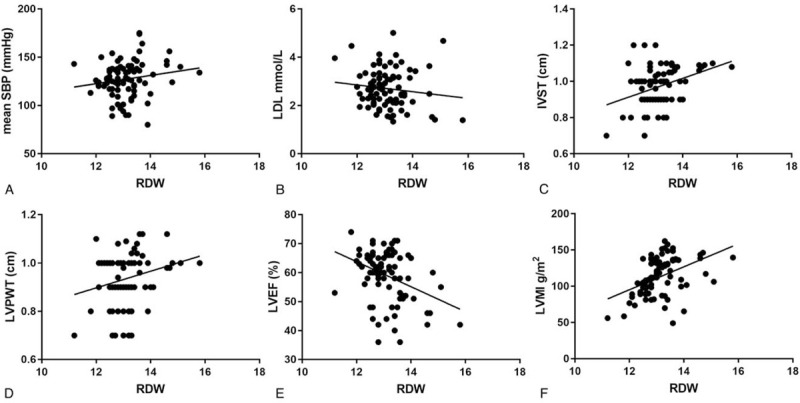
Pearson correlation tests between red blood cell distribution width (RDW) and other potential risk factors. (A) Correlation between RDW and mean systolic blood pressure (SBP) (*r* = 0.172, *P* > .05). (B) Correlation between RDW and low-density lipoprotein (LDL) (*r* = −0.129, *P* > .05). (C) Correlation between RDW and interventricular septal thickness (IVST) (*r* = 0.126, *P* < .05). (D) Correlation between RDW and mean left ventricular posterior wall thickness (LVPWT) (*r* = 0.058, *P* < .05). (E) Correlation between RDW and left ventricular ejection fraction (LVEF) (*r* = −0.130, *P* < .05). (F) Correlation between RDW and left ventricular myocardial mass index (LVMI) (*r* = 0.204, *P* < .05).

To determine the correlation between RDW and other potential risk factors, Pearson correlation coefficients were calculated in patients with AF (Fig. 1). IVST (*r* = 0.126, *P* < .05), LVPWT (*r* = 0.058, *P* < .05), and LVMI (*r* = 0.204, *P* < .05) had a positive relationship with RDW, while LVEF (*r* = –0.130, *P* < .05) had an inverse relationship with RDW. However, mean SBP and LDL were found to have no significant correlation with RDW in the entire study population (both *P* values >.05).

### 3.3 The risk factors for LVH in patients with AF

Additionally, multivariate logistic regression analysis was carried out to identify the potential risk factors for LVH in patients with AF. The results showed that RDW (odds ratio [OR] 4.891, 95% confidence interval [CI]: 1.693–14.132, *P* = .003) was an independent risk factor, while the concentration of LDL (OR 0.37, 95% CI: 0.17–0.83, *P* < .05) and LVEF (OR 0.88, 95% CI: 0.82–0.95, *P* < .05) were inversely associated with LVH in patients with AF. Regarding mean SBP, LVPWT, and IVST, there were no statistically significant differences between the 2 groups (all *P* values >.05) (Table 2).

**Table 2:**
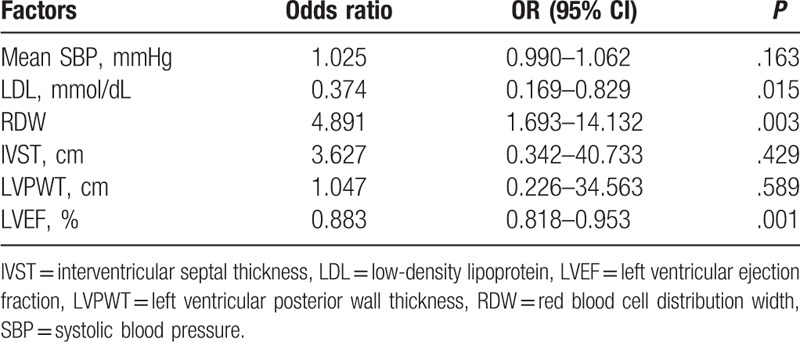
Multivariate logistic regression models analysis the risk factors.

### 3.4 Value of RDW for predicting LVH

**Figure 2:**
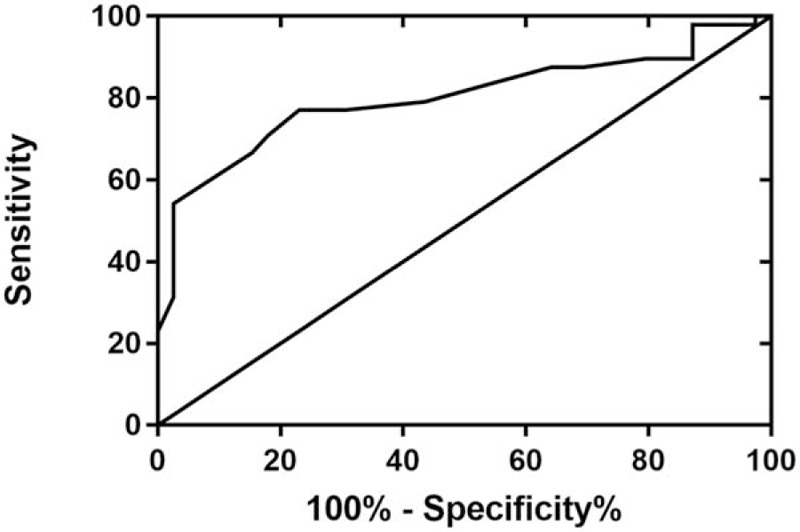
Receiver operating characteristic (ROC) curve of red blood cell distribution width (RDW). The ROC curve demonstrated the sensitivities and specificities of RDW for predicting the risk of left ventricular hypertrophy in patients with atrial fibrillation.

In patients with AF, ROC curves explored the value of RDW for predicting LVH. The area under the curve was 0.80 (95% CI: 0.71–0.90, *P* < .05) with a cut-off value of 13.05, and the RDW predicted LVH status among AF patients with a sensitivity of 72.1% and a specificity of 76.9% (Fig. 2).

## 4 Discussion

This study demonstrates the relationship between RDW and LVH in patients with AF. First, the main finding of our study indicates that RDW is significantly increased in AF patients with LVH compared with that in the patients in the non-LVH group. Second, several other conditions have been described as being associated with RDW. Third, multivariate logistic regression analysis suggested that RDW was an independent risk factor for LVH. Fourth, RDW >13.05 measured in patients who were recently diagnosed with AF had a 72.1% sensitivity and 76.9% specificity for predicting LVH.

RDW is a parameter of the variability in the size of circulating red blood cells measured by an automatic blood count instrument, and is traditionally used in laboratory hematology for the differential diagnosis of anemia.^[13,14]^ RDW has recently attracted increased attention as a marker of LVH in various cardiovascular disease states, including untreated essential hypertension, chronic kidney disease, and ischemic failure heart. Chen et al^[7]^ demonstrated that RDW was higher in untreated newly diagnosed hypertensive patients with LVH than in patients in the non-LVH group. The multiple logistic regression model indicated that patients with a higher RDW level were more likely to have LVH.^[7]^ A study that included 73 ambulatory patients with chronic kidney disease found that patients with advanced stage chronic kidney disease with left ventricular diastolic dysfunction were characterized by higher RDW levels than patients with advanced stage chronic kidney disease without left ventricular diastolic dysfunction.^[15]^ AlNajjar et al^[16]^ showed that the RDW was essential for evaluating the prognosis of patients with chronic heart failure, and its prognostic strength was comparable to that of NT-proBNP levels. A previous study aimed at assessing the prognostic value of RDW in post myocardial infarction patients with symptoms and typical signs of heart failure and with reduced LVEF showed that RDW was related to the presence of severe left ventricular dysfunction, with an accuracy of 61.4% (95% CI: 56.2%–66.4%) and 66.9% (95% CI: 61.8%–71.6%), using cut-off values of higher than 13.5% and 13.8%, respectively.^[17]^ In this study, the RDW predicted LVH status with a sensitivity of 72.1% and a specificity of 76.9%, based on a cut-off value of higher than 13.05%. Our result that the RDW value was independently associated with LVH was consistent with some points of previous reports that showed a significant association between RDW and echocardiographic parameters for the assessment of LV function in AF patients.

A possible explanation for the association between RDW and LVH might be the presence of oxidative stress in the body. Increased oxidative stress may inhibit the maturation of erythrocytes, resulting in the entry of immature erythrocytes into the general circulation, leading to an increase in the heterogeneity of peripheral erythrocyte morphology.^[14,18]^ Semba et al^[10]^ found that serum selenium was an independent predictor of RDW and could theoretically protect red cells from increased RDW by protecting erythrocytes from oxidative stress. This suggested that oxidative stress might be a potential biological mechanism for increased RDW. Ansari et al^[19]^ found that oxidative stress was associated with RDW by damaging the erythrocyte membrane, disturbing normal energy metabolism of erythrocytes, and shortening erythrocyte life span. Cave et al^[20]^ found that in cultured cardiomyocytes, cardiac hypertrophy caused by angiotensin II, endothelin-1, tumor necrosis factor (TNF), and pulsatile mechanical stretch was associated with intracellular reactive oxygen species production, which could be inhibited by antioxidants. The antioxidant and oxidant systems were imbalanced, and oxidative stress occurred when many reactive oxygen species were produced or antioxidants were depleted.^[21,22]^ Therefore, the activation of key mediators of metabolic regulation as well as the reduction in the activity or depletion of endogenous antioxidants promotes the increase in RDW.^[23]^

Some studies have demonstrated that RDW is a novel marker for chronic inflammation. Chronic inflammation was proposed to increase RDW, and persistent inflammation has been shown to be a principal pathophysiologic finding in individuals with heart failure and endothelial dysfunction.^[24]^ It has been confirmed that inflammatory cytokines such as TNF-α, interleukin (IL)-1β, and IL-6 desensitize bone marrow erythroid progenitors to erythropoiesis, inhibit red blood cell maturation and thereby promote anisocytosis, resulting in an increase in RDW.^[8]^ Pierce and Larson^[25]^ found that inflammatory cytokines affected iron metabolism and inhibited bone marrow, which caused an increase in RDW. The connection between chronic inflammation and LVH would be more significant as the disease progresses. This might also indicate that the RDW was a sensitive indicator to predict LVH from the other side. Semba et al^[10]^ investigated the values of serum antioxidants and inflammation in predicting RDW values in older women and found that patients in the higher quartiles of RDW might have a higher level of IL-6. However, the specific molecular mechanism of elevated RDW remains controversial.

Several limitations of our study should be noted. First, several markers of inflammation, such as C-reactive protein, IL-6, and TNF-α, were not evaluated. Second, one-time measurement of circulating red blood cells is another limitation in our study that increases the possibility of analytic drawbacks in regard to RDW measurements. Third, this is a retrospective study with a relatively small sample, which may have resulted in bias in the results of this study. Thus, a large sample of patients and a multicenter study are needed to verify the results of our study.

## 5 Conclusion

Based on the analysis, we established that approximately 44.8% of patients with AF developed LVH. Additionally, we speculated that RDW was associated with LVH in patients with AF.

## Acknowledgments

The authors would like to thank all the staff members at the First Affiliated Hospital of Nanchang University.

## Author contributions

**Conceptualization:** Hui-Ming Yao, Xiao-Lei Wang, Xiong Peng.

**Date curation and analysis:** Hui-Ming Yao, Xiao-Lei Wang.

**Investigation:** Xiong Peng, Shu-Yun Chen, Xuan Wan, Wei Zuo, Xin Gan.

**Writing – original draft**: Hui-Ming Yao, Xiao-Lei Wang, Xiong Peng.

**Writing – review & editing**: Hui-Ming Yao, Xiao-Lei Wang, Xiong Peng, Xin Gan.
